# Enhancing the efficacy of traditional Mongolian alcohol-spraying bone-setting therapy for surgical neck fractures of the humerus: the effects and mechanisms of muscular origin-insertion point massage therapy

**DOI:** 10.3389/fsurg.2025.1692036

**Published:** 2026-02-02

**Authors:** Changjiang Xie, Mengte Du, Xin Wang, Huqitu Xi, Tianhu Wei

**Affiliations:** 1 Graduate School of Inner Mongolia Medical University, Inner Mongolia Medical University, Hohhot City, Inner Mongolia, China; 2 Mongolian Medicine Orthopedics, Inner Mongolia International Mongolian Medicine Hospital, Hohhot, Inner Mongolia, China; 3 Affiliated Mongolian Medical Clinical College of Inner Mongolia Medical University, Hohhot, Inner Mongolia, China; 4 Bao Shi Zheng Orthopedic Department, Inner Mongolia International Mongolian Medicine Hospital, Hohhot, Inner Mongolia, China

**Keywords:** alcohol-spraying bone-setting therapy, hemorheology, humeral surgical neck fracture, Mongolian medicine, muscular origin-insertion point massage

## Abstract

**Background:**

Humeral surgical neck fractures (HSNF) are among the most common shoulder fractures, yet their treatment outcomes remain variable. Clinical observations suggest that combining traditional Mongolian alcohol-spraying bone-setting therapy with muscular origin-insertion point massage may accelerate fracture healing; however, the therapeutic efficacy and underlying mechanisms require further validation.

**Methods:**

A total of patients with HSNF were randomly allocated into two groups. The control group received Mongolian alcohol-spraying bone-setting therapy, while the experimental group received the same treatment plus muscular origin-insertion point massage. Clinical outcomes were assessed using the Constant-Murley and Neer shoulder function scores at multiple time points. To explore potential mechanisms, serum levels of soluble intercellular adhesion molecule-1 (sICAM-1), soluble vascular cell adhesion molecule-1 (sVCAM-1), platelet-derived growth factor (PDGF), insulin-like growth factor-1 (IGF-1), and hemorheological parameters (erythrocyte aggregation index, plasma viscosity) were measured.

**Results:**

From four weeks post-treatment onward, the experimental group exhibited significantly higher Constant-Murley and Neer scores compared with the control group (*p* < 0.05). Biochemical analysis showed that serum sICAM-1 was significantly lower in the experimental group beginning at two weeks (*p* = 0.02), whereas sVCAM-1 was reduced from four weeks (*p* < 0.01). In contrast, PDGF (*p* = 0.03) and IGF-1 (*p* = 0.04) were significantly elevated from four weeks onward in the experimental group. Moreover, erythrocyte aggregation index (*p* = 0.02) and plasma viscosity (*p* = 0.04) were significantly lower in the experimental group from four weeks post-treatment.

**Conclusion:**

The combination of muscular origin-insertion point massage with Mongolian alcohol-spraying bone-setting therapy yields favorable clinical outcomes, potentially by alleviating local inflammation and enhancing microcirculation, thereby promoting fracture healing. This integrative approach may represent a promising therapeutic strategy for the management of HSNF.

## Introduction

Humeral surgical neck fractures (HSNF) are among the most common proximal humeral injuries seen in clinical practice (1–3). These injuries are generally caused by direct or indirect trauma and typically occur 2–3 cm distal to the anatomical neck, above the insertion of the pectoralis major muscle. This region represents a transition zone from cancellous to cortical bone, rendering it structurally vulnerable. Clinically, patients with HSNF often present with localized swelling, severe pain, and marked limitation of upper limb mobility (4, 5). Displaced fractures may result in limb shortening and angular deformities. Additionally, some patients sustain neurovascular injuries, which significantly affect shoulder function and daily activities (6).

Current treatment strategies for HSNF include both conservative and surgical approaches (7, 8). Although many clinical studies have reported on these methods, there remains no unified treatment protocol widely accepted across institutions (9). Conservative management, commonly indicated for minimally displaced Neer type I and II fractures, avoids surgical trauma and preserves soft-tissue integrity and vascular supply, yet may be associated with prolonged immobilization, joint stiffness, and delayed functional recovery (10, 11). In contrast, surgical intervention provides more immediate anatomical stabilization but carries higher risks of soft-tissue injury and postoperative complications, limiting its applicability in elderly or medically complex patients (12–14).

Within this therapeutic landscape, Mongolian alcohol-assisted bone-setting therapy has developed as an alternative non-surgical approach grounded in traditional Chinese medicine principles. The technique uses a combination of dynamic and static maneuvers to achieve reduction while preserving soft-tissue structures, followed by the application of a specially designed splint that maintains fracture stability while allowing early joint movement. This method aims to promote fracture healing and reduce complications related to immobilization. Despite its long history of use in certain regions, treatment responses remain variable, and some patients continue to experience issues such as reduced muscle tone, adhesions, and delayed restoration of function (15–17), suggesting that additional supportive measures may be beneficial.

Muscle origin–insertion point massage therapy has recently been introduced as an adjunct to address these challenges. This technique specifically targets muscles closely related to shoulder mobility and pain, with the aim of improving muscle tone, enhancing limb function, and promoting local circulation. Although changes in inflammatory markers and growth factors—such as sICAM-1, sVCAM-1, VEGF, PDGF, and IGF-1—have been implicated in fracture repair and may offer mechanistic insights (18–21), clinical evidence regarding the combined application of Mongolian alcohol-assisted bone-setting therapy and origin–insertion point massage remains scarce.

Given these considerations, the present study was designed to evaluate whether the addition of muscle origin–insertion point massage therapy can enhance the therapeutic outcomes of Mongolian alcohol-assisted bone-setting treatment for HSNF. By integrating clinical assessment with relevant biological indicators, this work aims to provide evidence supporting a more effective and standardized conservative treatment strategy.

## Materials and methods

### Patients and grouping

This study enrolled 200 patients with HSNF who were admitted to our hospital between September 2020 and September 2023, irrespective of sex. The protocol was reviewed and approved by the Ethics Committee of the China Inner Mongolia International Mongolian Medicine Hospital (ethics approval number: 2020-006) and conducted in accordance with the principles of the Declaration of Helsinki (JBJS 79A:1089-1098, 1997). Patient confidentiality was strictly maintained in compliance with the Health Insurance Portability and Accountability Act (HIPAA) of the United States.

Inclusion criteria: 1. Clearly diagnosed with fresh closed HSNF, confirmed by x-ray, with fractures located in the surgical neck region, with or without angular deformities; 2. Age 30–70 years; 3. No significant pre-existing motor dysfunction, nerve injury, or limb deformities before the fracture; 4. Signed informed consent, with a preference for conservative treatment and willingness to comply with follow-up and clinical research.

Exclusion criteria: 1. Psychiatric disorders, severe infections, or organ failure (heart, liver, kidney, etc.); 2. Fractures with severe vascular or nerve injuries; 3. Pathological fractures caused by benign or malignant tumors, bone tuberculosis, or pyogenic osteomyelitis; 4. Open fractures or fractures that had previously undergone treatment; 5. Severe local swelling, extensive tension blisters, or other contraindications for Mongolian alcohol-spraying bone-setting therapy; 6. Patients unable to comply with follow-up or those lost to follow-up.

In this study, the criteria for detecting between-group differences were set at *α* = 0.05 and *β* = 0.20. To meet the requirements of a single-factor analysis and to minimize the risk of false-negative results due to insufficient sample size—while also avoiding excessive sample size that could increase loss to follow-up, complicate ethical approval, and impose unnecessary resource burdens—we determined the final sample size based on these considerations and the patient recruitment capacity of our institution. Accordingly, the total sample size was set at 100 participants. The patients were further randomized using the sealed-envelope method (Table 1). Throughout the clinical trial, both the treating clinicians and the outcome assessors remained blinded to the group assignments. After enrollment, patients were randomly divided into two groups (observation and control), with 100 cases in each group. The control group received Mongolian alcohol-spraying bone-setting therapy, while the experimental group additionally received muscular origin-insertion point massage therapy.

**Table 1:** Comparison of baseline characteristics between Two patient groups.

Group	Treatment group	Control group
Number of patients	50	51
Age^a^	61 (47.5–70)	69 (54–74)
Gender		
Female	35 (70.00%)	40 (78.43%)
Male	15 (30.00%)	11 (21.57%)
	25.1 ± 3.71	25.15 ± 4.04
Neer classification^b^		
Ⅰ	15 (30.00%)	16 (31.37%)
Ⅱ	29 (58.00%)	32 (62.75%)
Ⅲ	2 (4.00%)	2 (3.92%)
Ⅴ	4 (8.00%)	1 (1.96%)
Injured location		
Right side	24 (48.00%)	26 (50.98%)
Left side	26 (52.00%)	25 (49.02%)
Injury mechanism^b^		
Low-energy fall	46 (92.00%)	48 (94.12%)
High-energy trauma	4 (8.00%)	3 (5.88%)
Comorbidities		
Diabetes		
No	44 (88.00%)	42 (82.35%)
Yes	6 (12.00%)	9 (17.65%)
Hypertension^b^		
No	37 (74.00%)	38 (74.51%)
Yes	13 (26.00%)	13 (25.49%)
Coronary heart disease^b^		
No	49 (98.00%)	43 (84.31%)
Yes	1 (2.00%)	8 (15.69%)
Medication Use		
Loxoprofen Sodium		
Dispersible Tablets^b^		
Not use	47 (94.00%)	44 (86.27%)
Use	3 (6.00%)	7 (13.73%)
Ketorolac		
tromethamine^b^		
Not use	47 (94.00%)	46 (90.20%)
Use	3 (6.00%)	5 (9.80%)
Diclofenac sodium^b^		
Not use	46 (92.00%)	49 (96.08%)
Use	4 (8.00%)	2 (3.92%)
Methylprednisolone^b^		
Not use	49 (98.00%)	51 (100.00%)
Use	1 (2.00%)	0 (0.00%)
Ibuprofen		
Oral Solution^b^		
Not use	49 (98.00%)	51 (100.00%)
Use	1 (2.00%)	0 (0.00%)
Rivaroxaban^b^		
Not use	49(98.00%)	50(98.04%)
Use	1(2.00%)	1(1.96%)

a Median(Interquartile range, Q1–Q3), Wilcoxon Rank Sum test.

b Fisher’s precision probability test.

### Implementation of Mongolian alcohol-spraying bone-setting therapy

This therapy represents a classical treatment method that has been widely applied in Inner Mongolia, China, and validated through long-term clinical practice. The entire treatment process was performed in strict accordance with the established protocol of this therapy. All massage interventions in this study were performed by a single experienced Mongolian medical massage therapist, who has 15 years of clinical experience and holds formal teaching and supervisory qualifications in Mongolian manual therapy. The therapist consistently applied the same procedures and standards when treating all patients involved in this study. Specifically, patients were placed in a comfortable position conducive to fracture reduction. The therapist held alcohol in the mouth and sprayed it rapidly and extensively over the fracture site and surrounding area.

For abduction-type fractures, the surgeon applied traction to the proximal fragment while an assistant provided counter-traction to the distal fragment to correct displacement and overlap. During this maneuver, the surgeon stabilized the lateral aspect of the proximal fragment with both thumbs, applied outward traction with the other fingers, and adducted the elbow to achieve reduction and fixation.

For adduction-type fractures, reduction was achieved using an overhead abduction technique. The anterior portion of the affected area was pushed posteriorly while the patient's arm was gently pulled to correct the deformity. In cases of severe deformity, the arm was gradually elevated above the head, with the surgeon's thumbs stabilizing the distal fracture. In elderly patients or those with minor deformities, a soft pillow was placed under the axilla for additional support, and the arm was pressed downward to ensure fixation.

In patients with concomitant shoulder dislocation, fracture and dislocation reduction were performed simultaneously. For impacted or fissured fractures, appropriate reduction maneuvers were selected according to fracture morphology. Following reduction, anteroposterior and axillary x-rays were obtained to confirm restoration of the glenohumeral joint and to assess whether the fracture alignment met reduction standards.

After reduction, four small splints were applied. The anterior and posterior lateral splints extended approximately 2–3 cm beyond the shoulder joint and were positioned below the elbow joint. The inner short splint was padded with cotton at the proximal end, forming a mushroom-like shape. A long fastening strap was attached to the splint, and the anterior and posterior lateral splints were secured simultaneously. Bandaging was performed in three segments, with the upper segment wrapped in a figure-of-eight pattern to lock the shoulder splint and secured around the contralateral axilla. Splint tightness was adjusted according to the patient's range of motion, and a triangular bandage was applied to stabilize the reduction.

### Implementation of muscular origin-insertion point massage therapy

Based on the patient's pain distribution in the shoulder, elbow, forearm, and wrist, and taking into account the functional and innervation characteristics of each muscle group, specific muscular origin–insertion points were selected for massage. In particular, massage and percussion were applied to the shoulder, elbow, and wrist of the affected side, targeting muscles including the deltoid, supraspinatus, infraspinatus, pectoralis major, pectoralis minor, biceps brachii, and brachioradialis. The massage was performed until the patient experienced mild soreness accompanied by a distinct warm sensation at the affected site. This procedure was repeated 5–10 times per session, with each session lasting approximately 30 min, and was administered once daily.

### Neer shoulder function score assessment

Shoulder function was evaluated at baseline and at 1 week, 2 weeks, 4 weeks, and 8 weeks post-treatment using the Neer shoulder function score (22), which includes four parameters: pain (35 points), function (30 points), range of motion (25 points), and structural reduction (10 points). A total score of >89 points was considered excellent, 80–89 points satisfactory, 70–79 points unsatisfactory, and <70 points failure. All evaluators were blinded to the group allocation of the patients.

### Constan-Murley shoulder function score assessment

Shoulder function was also evaluated at baseline and at 1 week, 2 weeks, 4 weeks, and 8 weeks post-treatment using the Constan-Murley shoulder function score (22), which includes four parameters: shoulder pain (15 points), activities of daily living (20 points), range of motion (40 points), and abduction muscle strength recovery (25 points). A total score of 90–100 points was considered excellent, 80–89 points good, 70–79 points fair, and <70 points poor. All evaluators were blinded to the group allocation of the patients.

### Analysis of serum sICAM-1, sVCAM-1, PDGF, and IGF-1 Levels

Fasting venous blood samples (5 mL) were collected from all patients at baseline and at 1, 2, 4, and 8 weeks post-treatment. Serum levels of soluble intercellular adhesion molecule-1 (sICAM-1), soluble vascular cell adhesion molecule-1 (sVCAM-1), platelet-derived growth factor (PDGF), and insulin-like growth factor-1 (IGF-1) were quantified using enzyme-linked immunosorbent assay (ELISA). Blood samples were allowed to coagulate at room temperature for 30 min, followed by centrifugation at 1,000–2,000× g for 10 min at 4 °C. The resulting serum supernatant was aliquoted and stored at −80 °C until analysis. Commercial ELISA kits (Beyotime, China) were used to determine serum concentrations, with standard curves generated from the absorbance values at 450 nm of the standards. Sample concentrations were subsequently calculated based on the corresponding standard curves.

### Hemorheological index analysis

Fasting venous blood (5 mL) was collected at baseline and at 1 week, 2 weeks, 4 weeks, and 8 weeks post-treatment, with heparin anticoagulation. Hemorheological parameters, including the erythrocyte aggregation index and plasma viscosity, were measured using an automated blood rheometer (HT-100C, Hengtuo, China).

### Statistical analysis

Data were analyzed using SPSS Statistics 24.0 (IBM, USA). The Shapiro–Wilk test was used to assess the normality of numerical data. For non-normally distributed data, the Mann–Whitney *U*-test was applied for group comparisons. For normally distributed data, the *F*-test was first conducted to evaluate the homogeneity of variances. When variances were homogeneous, an independent-samples *t*-test was performed. In cases of heterogeneity of variances, Welch's *t*-test was used. A *p*-value of <0.05 was considered statistically significant.

## Results

Muscle Origin-Insertion Point Massage Significantly Accelerated and Improved Shoulder Joint Function Recovery in Patients Receiving Mongolian Alcohol-spraying Bone-setting Therapy.

Effectiveness assessment (Tables 2, 3) demonstrated that the experimental group, which received muscular origin–insertion point massage in addition to Mongolian alcohol-spraying bone-setting therapy, exhibited significantly higher Constant-Murley and Neer shoulder function scores early in the treatment course compared with the control group, with this advantage persisting throughout the study period. Specifically, at 4 weeks post-treatment, the Neer shoulder function score in the experimental group (81.2 ± 6.5) was significantly higher than in the control group (69.1 ± 4.9; *p* = 0.027), and this superiority was maintained at weeks 6 and 8.

**Table 2:** Neer shoulder function scores at different time points.

Time point	Experimental group	Control group	*T*-value	*P*-value
Pre-treatment	62.2 ± 3.4	61.2 ± 3.9	0.298	0.812
2 weeks post-treatment	71.4 ± 8.3	62.4 ± 8.1	6.019	0.094
4 weeks post-treatment	81.2 ± 6.5	69.1 ± 4.9	10.125	0.027
6 weeks post-treatment	84.3 ± 9.5	78.4 ± 8.8	11.782	<0.01
8 weeks post-treatment	91.2 ± 5.8	82.1 ± 6.6	13.330	<0.01

Independent samples *t*-test; Data are presented as mean ± standard deviation.

**Table 3:** Constan-Murley shoulder function scores at different time points.

Time point	Experimental group	Control group	*T*-value	*P*-value
Pre-treatment	48.6 ± 3.7	47.4 ± 6.4	0.571	0.883
2 weeks post-treatment	62.4 ± 6.3	60.7 ± 8.8	1.894	0.071
4 weeks post-treatment	79.1 ± 9.4	68.4 ± 8.3	10.451	<0.01
6 weeks post-treatment	86.7 ± 6.2	77.2 ± 7.0	12.437	<0.01
8 weeks post-treatment	93.4 ± 5.3	81.6 ± 6.1	14.778	<0.01

Independent samples *t*-test; Data are presented as mean ± standard deviation.

Similarly, the Constant-Murley score at week 4 in the experimental group (79.1 ± 9.4) was significantly higher than that of the control group (68.4 ± 8.3; *p* < 0.01), with subsequent scores following a trend consistent with that observed for the Neer scores. By week 8, the mean scores for both assessment tools in the experimental group exceeded 90, indicating excellent functional recovery. These findings suggest that the addition of muscular origin–insertion point massage accelerates therapeutic efficacy and promotes high-quality restoration of shoulder function.

Muscle Origin-Insertion Point Massage Significantly Improved the Elevated Inflammatory Levels in Patients Receiving Mongolian Alcohol-spraying Bone-setting Therapy.

Elisa testing (Tables 4, 5) showed that sVCAM-1 levels in the experimental group were significantly lower than in the control group starting at week 4 post-treatment (*p* < 0.01), with this difference sustained in subsequent measurements. The difference in sICAM-1 levels appeared earlier. Specifically, at week 2 post-treatment, the experimental group's sICAM-1 levels (217.02 ± 18.60) were significantly lower than the control group (228.42 ± 14.85), and this lower trend continued at weeks 4, 6, and 8, suggesting that muscle origin-insertion point massage further improves the inflammatory abnormalities associated with humerus surgical neck fractures.

**Table 4:** sICAM-1 levels at different time points.

Time point	Experimental group	Control group	*T*-value	*P*-value
Pre-treatment	167.49 ± 10.97	168.78 ± 10.74	−0.59	0.883
2 weeks post-treatment	217.02 ± 18.60	228.42 ± 14.85	−10.87	0.02
4 weeks post-treatment	229.16 ± 16.34	270.14 ± 19.80	−39.57	<0.01
6 weeks post-treatment	239.18 ± 15.39	298.04 ± 16.97	−49.72	<0.01
8 weeks post-treatment	250.14 ± 22.03	310.15 ± 21.06	−61.74	<0.01

Independent samples *t*-test; Data are presented as mean ± standard deviation.

**Table 5:** sVCAM-1 levels at different time points.

Time point	Experimental group	Control group	*T*-value	*P*-value
Pre-treatment	418.05 ± 27.46	416.88 ± 29.47	1.89	0.57
2 weeks post-treatment	468.08 ± 37.58	472.39 ± 40.21	−6.47	0.28
4 weeks post-treatment	479.31 ± 47.87	530.71 ± 44.13	−24.87	<0.01
6 weeks post-treatment	502.78 ± 45.79	553.76 ± 41.02	−66.31	<0.01
8 weeks post-treatment	520.34 ± 39.64	590.18 ± 34.23	−74.15	<0.01

Independent samples *t*-test; Data are presented as mean ± standard deviation.

Muscle Origin-Insertion Point Massage Significantly Enhanced Nutritional Factor Levels Associated with Fracture Healing in Patients Receiving Mongolian Alcohol-spraying Bone-setting Therapy.

In terms of PDGF levels (Table 6), the experimental group (210.37 ± 12.46) showed a significant increase compared to the control group (187.27 ± 14.54, *p* = 0.03) by week 4 post-treatment, and this advantage remained significant at weeks 6 and 8. Similarly, the trend for IGF-1 (Table 7) was comparable to that of PDGF. The experimental group showed significantly higher IGF-1 levels at week 4 (291.33 ± 20.34 vs. 283.81 ± 21.94, *p* = 0.04), with this difference maintained in subsequent weeks.

**Table 6:** PDGF levels at different time points.

Time point	Experimental group	Control group	*T*-value	*P*-value
Pre-treatment	108.97 ± 13.45	110.03 ± 11.98	0.87	0.66
2 weeks post-treatment	158.96 ± 17.23	149.39 ± 12.54	8.37	0.23
4 weeks post-treatment	210.37 ± 12.46	187.27 ± 14.54	16.97	0.03
6 weeks post-treatment	240.18 ± 18.29	205.88 ± 18.89	35.28	<0.01
8 weeks post-treatment	260.78 ± 27.95	211.27 ± 18.02	57.09	<0.01

Independent samples *t*-test; Data are presented as mean ± standard deviation.

**Table 7:** IGF-1 levels at different time points.

Time point	Experimental group	Control group	*T*-value	*P*-value
Pre-treatment	218.84 ± 12.74	219.76 ± 11.87	0.67	0.88
2 weeks post-treatment	257.79 ± 18.45	250.37 ± 19.64	7.12	0.16
4 weeks post-treatment	291.33 ± 20.34	283.81 ± 21.94	11.42	0.04
6 weeks post-treatment	329.55 ± 19.08	297.02 ± 18.74	35.17	<0.01
8 weeks post-treatment	351.33 ± 22.87	310.16 ± 20.96	45.08	<0.01

Independent samples *t*-test; Data are presented as mean ± standard deviation.

Muscle Origin-Insertion Point Massage Significantly Improved Blood Circulation in Patients Receiving Mongolian Alcohol-spraying Bone-setting Therapy.

Analysis of the red blood cell aggregation index (Table 8) revealed no significant differences between the experimental and control groups prior to treatment and at 2 weeks post-treatment. However, by week 4, the experimental group demonstrated a significantly lower red blood cell aggregation index compared with the control group (3.64 ± 0.40 vs. 5.77 ± 0.48; *p* = 0.02), and this difference persisted throughout the study period. Similarly, plasma viscosity (Table 9) showed a significant reduction in the experimental group starting at week 4 (1.29 ± 0.18 vs. 1.80 ± 0.21; *p* = 0.04), with continued significant differences observed at weeks 6 and 8. Collectively, these findings indicate that the addition of muscular origin–insertion point massage further enhances blood circulation during the treatment of humeral surgical neck fractures with Mongolian alcohol-spraying bone-setting therapy.

**Table 8:** Red blood cell aggregation Index at different time points.

Time point	Experimental group	Control group	*T*-value	*P*-value
Pre-treatment	4.11 ± 0.37	4.10 ± 0.55	0.84	0.74
2 weeks post-treatment	4.02 ± 0.51	4.95 ± 0.45	−1.03	0.45
4 weeks post-treatment	3.64 ± 0.40	5.77 ± 0.48	−2.29	0.02
6 weeks post-treatment	3.01 ± 0.33	6.37 ± 0.64	−4.21	<0.01
8 weeks post-treatment	2.67 ± 0.44	6.88 ± 0.67	−5.13	<0.01

Independent samples *t*-test; Data are presented as mean ± standard deviation.

**Table 9:** Plasma viscosity at different time points.

Time point	Experimental group	Control group	*T*-value	*P*-value
Pre-treatment	1.53 ± 0.24	1.55 ± 0.20	−0.10	0.76
2 weeks post-treatment	1.39 ± 0.20	1.69 ± 0.18	−0.51	0.37
4 weeks post-treatment	1.29 ± 0.18	1.80 ± 0.21	−0.54	0.04
6 weeks post-treatment	1.20 ± 0.20	1.89 ± 0.18	−0.69	<0.01
8 weeks post-treatment	1.09 ± 0.15	1.97 ± 0.23	−1.03	<0.01

Independent samples *t*-test; Data are presented as mean ± standard deviation.

## Discussion

Current studies have shown that the factors influencing fracture healing can be categorized into physical factors and intrinsic body factors. Physical factors, such as the location and severity of the fracture, have a more direct impact on fracture healing (23–25). Intrinsic body factors encompass hematologic and serological parameters, including the red blood cell aggregation index, erythrocyte rigidity index, plasma viscosity, and serum levels of sICAM-1, sVCAM-1, PDGF, and IGF-1. Fracture healing is a highly complex and organized biological repair process, influenced by blood circulation, biochemical and biomechanical regulation, and micro-nutrient availability. Delayed fracture healing carries considerable risks, including prolonged recovery, increased likelihood of reoperation, and reduced patient quality of life.

Mongolian bone-setting therapy has been shown to stimulate extracellular matrix synthesis, promote osteoblast proliferation, inhibit osteoclast differentiation, and enhance growth factor secretion, thereby facilitating fracture healing and preventing delayed union. In this study, patients treated with Mongolian alcohol-spraying bone-setting therapy exhibited continuous improvements in shoulder function scores throughout the treatment period. By week 6, scores approached near-satisfactory levels (approximately 80 points), with further improvement observed by week 8, demonstrating the fundamental efficacy of the therapy. Notably, when muscular origin–insertion point massage was added, patients reached the same functional level by week 4 rather than week 6, and scores ultimately achieved excellent levels by the end of follow-up. These findings indicate that the addition of muscular origin–insertion point massage not only accelerates rehabilitation but also promotes a more complete and robust recovery of shoulder function. Compared to recent studies on surgical and non-surgical treatments for proximal humeral fractures (26, 27), the recovery speed and final outcome in this study were superior.

In this study, we specifically investigated the roles of sICAM-1, sVCAM-1, PDGF, and IGF-1. Our findings showed that the levels of these factors progressively increased over time, likely reflecting activation of the body's repair mechanisms in response to bone and soft tissue injury, which stimulates both the inflammatory response and the secretion of repair-related mediators. However, excessive elevation of inflammatory factors can impede the healing process and compromise the final quality of bone repair. Thus, maintaining a balance between inflammation and repair is essential.

Follow-up analyses indicated that, although the overall temporal trend of increasing factor levels was not fundamentally altered by the addition of muscular origin–insertion point massage, the experimental group exhibited significant differences compared with the control group. Specifically, inflammatory markers sICAM-1 and sVCAM-1 were significantly lower in the experimental group during the early stages of treatment (weeks 2 and 4), whereas repair-associated growth factors PDGF and IGF-1 increased more rapidly and to a greater extent. These results suggest that muscular origin–insertion point massage not only helps mitigate excessive inflammatory responses but also enhances the expression of growth factors critical for bone repair, thereby potentially facilitating faster and higher-quality recovery of humeral surgical neck fractures. Given that upregulated inflammatory factors could also cause damage to various organs, including the lungs and liver (28, 29), the overall benefits of the new treatment approach in this study are substantial. It is noteworthy that, based on efficacy assessments during the first four weeks, the combination of massage and bone-setting therapy did promote functional recovery in patients. However, from the perspective of cytokine levels, although significant differences in ICAM-1 and VCAM-1 were observed during this period, the magnitude of change was limited, particularly for ICAM-1, where the difference was almost negligible. This suggests that changes in these factors may not be the sole mechanism underlying clinical efficacy, or that, even if they represent a primary mechanism, their effects may not manifest immediately or directly at the clinical level. The precise and comprehensive mechanisms require further investigation in future studies.

Mongolian medicine suggests that tissue swelling and pain following fractures are primarily due to the trauma-induced “He Yi” and the blockage of blood flow. “He Yi” is a disease entity in Mongolian medicine, which is believed to cause dysregulation of physiological functional dynamics, thereby affecting the overall circulation of energy and nutrients in the body. As such, Mongolian medicine emphasizes a holistic approach and dialectical treatment based on the regulation of “He Yi” blood to improve blood circulation, reduce swelling, alleviate pain, and promote fracture healing. In this study, we used plasma viscosity and red blood cell aggregation index as markers to compare the effects of muscle origin-insertion point massage on blood rheology. The results showed that in Mongolian alcohol-spraying bone-setting therapy, due to a lack of focus on local blood supply recovery, the red blood cell aggregation index and plasma viscosity increased over time. This aligns with the general pathophysiological understanding that local blood supply worsens after a fracture (30). However, after adding muscle origin-insertion point massage, no significant changes were observed in the blood parameters during the first two weeks. By week 4, both plasma viscosity and red blood cell aggregation index were significantly lower in the experimental group than in the control group, and this trend continued. These findings suggest that massage targeting the muscle origin and insertion points may positively influence local blood circulation at the fracture site, which could be a key mechanism for facilitating faster and more effective recovery of shoulder joint function after fracture. In addition, the potential therapeutic mechanisms of this approach may include the enhancement of mechanical signaling and lymphatic drainage, promoting interstitial fluid flow and facilitating the removal of metabolic waste and regulation of local inflammation (31). The potential effects mediated via proprioceptive neuromuscular pathways should not be overlooked, as they may enhance neural activation and coordinated control of the affected muscle groups, improve joint kinematics, and prevent muscle atrophy (32).

However, it is worth noting that although muscle origin-insertion point massage showed good effects in improving blood supply, this therapy requires a high degree of patient compliance and may involve some discomfort, which represents a therapeutic threshold. Nonetheless, considering the comprehensive benefits for fracture rehabilitation, we believe that the addition of muscle origin-insertion point massage to Mongolian alcohol-spraying bone-setting therapy is essential. Previous studies have followed up patients with proximal humeral fractures, with 52.4% classified as Neer type I and II, who received conservative treatment (33). At the final follow-up, the mean Constant-Murley Shoulder Function Score was 95. In the present study, postoperative shoulder function in the experimental group gradually improved during rehabilitation, reaching 93.4 ± 5.3 at the final follow-up, which is comparable to the reported level. Considering that our follow-up period was shorter than that of the previous study, the postoperative efficacy of the method proposed in this study can be regarded as acceptable. However, given the relatively small sample size and the limited diversity of patient sources in our study, it is not possible to conclude that this method has achieved an internationally advanced or superior level of efficacy.

This study has certain limitations. First, we did not record common indicators such as complications and fracture healing time but instead used shoulder joint function scores as a surrogate. This was because the primary aim of Mongolian alcohol spraying bone-setting therapy is to promote the functional rehabilitation of the affected limb and improve the patient's quality of life, rather than simply achieving bone union or preventing complications. Therefore, we directly used shoulder joint function scores, which represent the final indicator of shoulder function, to evaluate the therapy's effectiveness. Second, this was a single-center study. Although the total sample size is reasonable, the representativeness of the sample still needs improvement. Future studies will involve multi-center research with a wider range of case samples to derive more comprehensive conclusions. Third, since our hospital primarily practices traditional Mongolian medical therapies, we do not have standard surgical cases (open reduction and internal fixation with plating) for the above fractures. Consequently, direct comparative data with standard surgical treatments are unavailable. Comparisons can only be made in parallel with relevant studies conducted at other domestic and international institutions.

## Conclusion

Muscular origin–insertion point massage therapy may further enhance the therapeutic efficacy of traditional Mongolian alcohol-spraying bone-setting therapy in the management of humeral surgical neck fractures. This combined approach appears to facilitate fracture healing by attenuating local inflammation and improving blood circulation, representing a novel, non-surgical strategy for the treatment of fractures in various anatomical regions.

## Data Availability

The original contributions presented in the study are included in the article/Supplementary Material, further inquiries can be directed to the corresponding author.
